# Efficiency Comparison of Public Hospitals under Different Administrative Affiliations in China: A Pilot City Case

**DOI:** 10.3390/healthcare9040437

**Published:** 2021-04-08

**Authors:** Gang Yin, Chaoyi Chen, Lijun Zhuo, Qingjing He, Hongbing Tao

**Affiliations:** Department of Health Administration, School of Medicine and Health Management, Tongji Medical College, Huazhong University of Science and Technology, Wuhan 430000, China; yingang20@hust.edu.cn (G.Y.); chaoyi_chen@hust.edu.cn (C.C.); lijunzhuo@hust.edu.cn (L.Z.); heqingjing20@hust.edu.cn (Q.H.)

**Keywords:** public hospital efficiency, efficiency comparison, data envelopment approach

## Abstract

This study seeks to measure the efficiency disparity and productivity change of tertiary general public hospitals in Wuhan city, central China from the perspective of administrative affiliations by using panel data from 2013 to 2017. Sample hospitals were divided into three categories, namely provincial hospitals, municipal hospitals, and other levels of hospitals. Data envelopment analysis with bootstrapping technique was used to estimate efficiency scores, and a sensitive analysis was performed by varying the specification of model by considering undesirable outputs to test robustness of estimation, and efficiency evolution analysis was carried out by using the Malmquist index. The results indicated that the average values of provincial hospitals and municipal hospitals have experienced efficiency improvement over the period, especially after the initiation of Pilot Public Hospital Reform, but hospitals under other affiliations showed an opposite trend. Meanwhile, differences of administrative subordination in technical efficiency of public hospitals emerged, and the disparity was likely to grow over time. The higher efficiency of hospitals affiliated with municipality, as compared with those governed by province and under other administrative affiliations, may be attributed to better governance and organization structure.

## 1. Introduction

Public medical institutions are considered to be an essential part of a country’s health service system worldwide. In recent years, efficiency of public hospitals has become a concern for policymakers and researchers dealing with health expenditure growth in both developed and developing countries [1,2,3,4,5,6]. In China, this issue has long drawn scholars’ attention, and several articles have been published [7,8,9,10,11,12,13,14,15]. Generally, Chinese public hospitals are classified according to three standards, namely service capacity (i.e., primary, secondary, and tertiary hospitals), service items (i.e., general and specialized hospitals), and administrative subordination by government agencies in accordance with regulations. However, a great number of studies only involve the first two classification criteria, but the last one has not been fully explored. Although public hospitals now enjoy considerably more autonomy regarding their revenues than during the planned economy era, policies for the public health sector still face certain constraints left over by government hierarchy [16,17]. In light of classification based on administrative subordination, the ownership of public hospitals in China can be roughly divided into two categories: government-owned and public institution-owned. Among them, government-owned hospitals can be mainly divided into three types, namely county, municipal, and provincial hospitals. They are consistent with the administrative level of government hierarchy in China (county, city, and province). As a small part of public hospitals, public institution-owned hospitals are often generally inferior in administrative status to their government-owned counterparts, but they have higher autonomy and independence in governance. On the one hand, the classification based on administrative subordination in public hospitals is in line with the system of public institution in China. It determines that governments have high governance over public facilities in human resources, financial budget, and medical business [16]. On the other hand, degrees of different governmental levels’ involvement in a specific public medical institution are not exactly the same. For instance, senior leaders of public hospitals with a higher administrative status will be able to be assigned and moved directly by higher levels of government. Consequently, it is reasonable to assume that there are certain gaps in efficiency among public hospitals under different relationships of administrative subordination. It remains to be seen whether public hospitals with higher administrative status are more efficient. 

In the past decade, the Chinese government has made a series of efforts to improve the condition of difficulty and high cost in terms of getting medical service in the nation. The New Medical and Health System Reform (NMHSR), which aims to guarantee fair access to basic medical and health service and was launched in 2009, has been a great success, partly improving residents’ accessibility to medical care [18,19,20]. As one of the five key pillars in the 2009 project, Pilot Public Hospital Reform (PPHR) was popularized in 2011 by the Chinese government. It is committed to implementing a new version of medical service prices, canceling drug price addition, and increasing financial subsidies in public health facilities, with focuses on operation efficiency, service quality, essential functions, and social responsibilities of medical units [21,22,23]. Wuhan (WH), as a representative city in central China and the capital of Hubei province, was identified as a member of the third cluster of PPHR in 2014. Undoubtedly, identifying the efficiency status of local public health sector can help design policy measures by highlighting factors which policymakers can act on. 

In terms of literature for hospital efficiency, the Data Envelopment Analysis (DEA) method has been widely used by scholars around the world when measuring efficiency and productivity of healthcare units and large-scale hospitals in particular. From the perspective of a research framework, relevant research work can be roughly divided into three categories: 

(1) Research evaluating facilities’ efficiency in the regional or cross-regional level. Area classification methods for administrative division [24,25,26,27,28,29,30,31], geographical location [32,33], and economic development level [34,35] are frequently used. (2) Research comparing the efficiency variance among different types of units. Public and private hospitals [36,37,38,39,40,41], and profitable and non-profit hospitals [42,43,44] are often compared, which is especially obvious in research by European and American scholars. Comparison in diverse hospital management systems [45,46] and scales of hospital [37,47] have also been adopted in recent years. (3) Research estimating the efficiency difference of hospitals before and after an event occurs, such as economic crisis [47,48], system reform [49,50,51,52,53,54,55], competitive context [56,57], institutions merger [58], project implementation [59,60], or privatization [61].

Through the aforementioned literature review, we can draw a conclusion that studies of efficiency comparison among public hospitals under different administrative subordination are still insufficient. For the framework of this research, 25 public hospitals affiliated with three levels of administrative organs were selected in WH during 2013–2017. DEA–Malmquist models were applied along with the bootstrapping method used for correction of efficiency values. This research sheds light on public hospital governance mode (i.e., autonomous or centralized) from the perspective of operational efficiency. The results of this study may help policymakers better understand efficiency changes among hospitals with different affiliations. It is conducive to the formulation of health reform policies in China. 

The rest of the study is organized as follows. Section 2 introduces the theoretical basis of analysis methods used, and explains selection of samples, indicators, model specifications, and analysis tools. Section 3 reports results of descriptive statistics, technical efficiency, and Malmquist index. Section 4 discusses findings from the aspects of efficiency variance, average efficiency, efficiency distribution, and productivity indices. Section 5 concludes those findings and clarifies limitations.

## 2. Materials and Methods

### 2.1. Data Envelopment Analysis of Hospital Efficiency in China

At present, the most common methods to evaluate the efficiency of a healthcare system are DEA, Stochastic Frontier Analysis (SFA), and Ratio Analysis (RA). DEA method is a typical nonparametric approach in efficiency estimation developed by Charnes, Cooper, and Rhodes in 1978, which does not require relative price information and a specific functional form for a production possibility frontier [62,63]. Meanwhile, the procedure of applying DEA considers a multidimensional perspective of the input and output for the healthcare sector [43]. Therefore, the DEA method is widely used in the efficacy estimation of medical treatment units. 

The concept of Technical Efficiency (TE), developed by Farrell in 1957, describes the capacity of a Decision-Making Unit (DMU) to produce the maximum amount of output from a given amount of input or, alternatively, to produce a given output with a minimum quantity of input [64,65]. As for the hospital sector, TE can be represented as producing a given level of medical service outputs with the least medical resource inputs. The area surrounded by the curve formed by DMUs, which composes the efficiency frontier, envelops the relatively inefficient DMUs. TE scores range from 0 to 1, respectively representing inefficiency and full efficiency. Generally, there are two orientations in the process of DEA, namely input orientation and output orientation. If DMU can freely adjust the number or proportion of its input indicators according to the needs of the market, it is suitable to adopt the input orientation model. However, it is hard for leaders in Chinese public hospitals to decide the number of doctors or nurses on their own. At the same time, the phenomenon of difficulty in getting medical service is common in Chinese public medical institutions, and the supply of medical services is not adequate for demand. Therefore, output-orientated Constant Returns to Scale (CRS) DEA method was adopted to obtain the TE scores for each healthcare sector in this study. [54] Suppose there are *n* DMUs’ TE (*DMU_j_*, *j* = 1, 2, …, *n*) need to be measured, and each DMU has *m* inputs (*x_i_*, *i* = 1, 2, …, *m*) and *q* outputs (*y_r_*, *r* = 1, 2, …, *q*). Please note that the weight of inputs and outputs as *v_i_* (*i* = 1, 2, …, *m*) and *u_r_* (*r* = 1, 2, …, *q*) respectively. The DMU currently measured was noted as *DMU_k_*. The output orientation Charnes Cooper Rhodes (CCR) model can be described as the following formula:

$$\mathrm{Minimize}:\;\overset{m}{\underset{i=1}{\sum }}v_{i}x_{\mathrm{ik}}\;\mathrm{Subject}\;\mathrm{to}:\;\overset{q}{\underset{r=1}{\sum }}u_{r}y_{\mathrm{rj}}-\overset{m}{\underset{i=1}{\sum }}v_{i}x_{\mathrm{ij}}\;\leq \;0\;\overset{q}{\underset{r=1}{\sum }}u_{r}y_{\mathrm{rk}}\;=\;1\;v\;\geq \;0;\;u\;\geq \;0\;i=1,\;2,\;\cdots ,\;m;\;r=1,\;2,\;\cdots ,\;q;\;j=1,\;2,\;\cdots ,\;n$$

Its dual model can be described as the following formula:

$$\mathrm{Maximize}:\;\varphi \;\mathrm{Subject}\;\mathrm{to}:\;\overset{n}{\underset{j=1}{\sum }}\lambda_{j}x_{\mathrm{ij}}\leq {\;x}_{\mathrm{ik}}\;\overset{n}{\underset{j=1}{\sum }}\lambda_{j}y_{\mathrm{rj}\;}\;\geq {\;\varphi y}_{\mathrm{rk}}\;\lambda \;\geq \;0\;i=1,\;2,\;\cdots ,\;m;\;r=1,\;2,\;\cdots ,\;q;\;j=1,\;2,\;\cdots ,\;n$$

Dual model means that each output can measure inefficiency with equal proportion growth based on the fixed input. Hence, it is called output-orientated CCR model. The optimal solution of the model is φ^*^. Under the condition of no increase of inputs, the maximum proportion of outputs growth of *DMU_k_* is φ^*^ − 1. The larger φ^*^ is, the greater the output can be increased and the lower the efficiency is. 1/φ^*^ was used to represent efficiency score since φ^*^ ≥ 1 [66].

### 2.2. Bias Correction of Efficiency with Bootstrapping Method

Based on the application of the classic DEA model, bootstrapping method is a popular statistical method in modern nonparametric statistics put forward by Efron in 1979, which performs interval estimation via estimating the variance of statistics and adopting repeated sampling to simulate the data generation process [67]. Meanwhile, this method approximately obtains the sample distribution and variance of the original estimator by using the original estimator in the simulation sample [68,69,70]. The bootstrapping method can be divided into the following steps:

Step 1: The original scores $\;\hat{\theta }$ of each ${\mathrm{DMU}}_{k}\;(k=1,\;2,\;\cdots ,\;n)\;$ was calculated by traditional DEA model. Then extracting a naive sample ${\hat{\theta }}^{b}\;$ of the scale *n* by bootstrapping method. Where b (1, 2,⋯, B) denotes the number of iterations of bootstrap sampling:

$${\hat{\theta }}^{b}=\left\{{\hat{\theta }}_{k}^{b}|k=1,\;2,\;\cdots ,\;n\right\}$$

Step 2: The Kernel density estimation method was used to smooth the samples obtained by naive bootstrap to get ${\bar{\theta }}^{b}$. The input indexes $x_{k}=(k=1,\;2,\;\cdots ,\;n)$ of the original sample were modified according to ${\bar{\theta }}^{b}$. The adjusted indexes were as follows:

$$x_{k}^{b}=\left({\hat{\theta }}_{k}{/\bar{\theta }}_{k}^{b}\right)x_{k}\;\left(k=1,\;2,\;\cdots ,\;n\right)$$

According to the bootstrap adjusted inputs and initial outputs as new samples, the traditional DEA method was used to recalculate the efficiency value ${\tilde{\theta }}^{b}$ as follows:

$${\tilde{\theta }}^{b}=\left\{{\tilde{\theta }}_{k}^{b}|k=1,\;2,\;\cdots ,\;n\right\}$$

Step 3: ${\tilde{\theta }}_{k}^{b}$ were obtained after repeating steps 1 and 2 with *B* times. The bias, corrected efficiency scores ${\tilde{\theta }}_{k}$ and the confidence interval (with the confidence level *α*) can be described as the following formula:

$${\mathrm{Bias}}_{k}=E\left({\tilde{\theta }}_{k}^{b}\right){-\hat{\theta }}_{k}=\frac{1}{B}\overset{B}{\underset{b=1}{\sum }}{\tilde{\theta }}_{k}^{b}{-\hat{\theta }}_{k}\;{\tilde{\theta }}_{k}{=\hat{\theta }}_{k}{-\mathrm{Bias}}_{k}{=2\hat{\theta }}_{k}-\frac{1}{B}\overset{B}{\underset{b=1}{\sum }}{\tilde{\theta }}_{k}^{b}\;b_{\alpha /2}{+\tilde{\theta }}_{k}\leq {\bar{\tilde{\theta }}}_{k}\leq {\tilde{\theta }}_{k}{+a}_{\alpha /2}$$

In this research, bootstrapping method was introduced into DEA model and corrected original DEA efficiency value by taking the influence of interference factors with 2000 replications into account. 

### 2.3. Estimation of Malmquist Index for Productivity Change

Malmquist Index (MI) measures the change of productivity by calculating the geometric mean of the productivity indexes between *t* and *t +* 1 period (i.e., Adjacent Malmquist). The formula can be expressed as follows:

$$\mathrm{MI}\left(x^{t}{,\;y}^{t}{,\;x}^{t+1}{,\;y}^{t+1}\right)\;=\;[\frac{D^{t}\left(x^{t+1}{,\;y}^{t+1}\right)}{D^{t}\left(x^{t}{,\;y}^{t}\right)}\times \frac{D^{t+1}\left(x^{t+1}{,\;y}^{t+1}\right)}{D^{t+1}\left(x^{t}{,\;y}^{t}\right)}{]}^{\frac{1}{2}}$$

where *x* denotes the input indexes, *y* denotes the output indexes, $D^{t}\left(x^{t}{,\;y}^{t}\right)$ is defined as the output distance function, and MI measures the total productivity changes between *t* and *t* + 1 period. [30] When *MI* > 1, it signifies increased productivity; when *MI* < 1, it signifies declined productivity; when *MI* = 1, it signifies constant productivity. The change can be decomposed into Technical Efficiency Change (EC) and Technological Change (TC) as follows:

MI = EC × TC

### 2.4. Study Population

The data used in this study was extracted from the Wuhan Health and Family Planning Yearbook (2014-2018). The sample under this study was all tertiary general public hospitals in WH. By considering the good comparability between samples, specialized hospitals, maternal and child health hospitals, traditional Chinese medicine hospitals, and army hospitals, and those affiliations and levels that were altered during the analyzed period were excluded. Finally, 25 public hospitals were included in the study. All the selected hospitals are officially classified as tertiary general public hospitals to meet the basic requirement in applying the DEA approach, namely the homogeneity of DMUs. 

More specifically, the National Health Administration had strict requirements on the Evaluation of Tertiary General Hospitals, including number of beds, department settings, number of medical staff, and medical equipment, which could ensure the similarity of sample hospitals in this research. 

**Figure 1:**
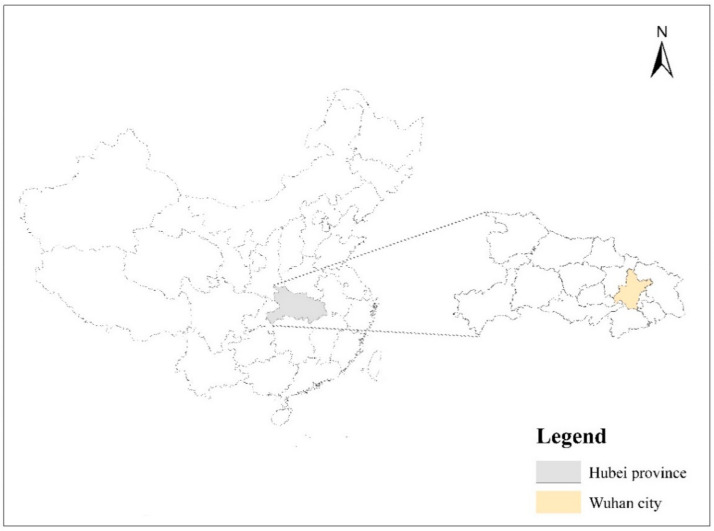
The geographical location of Wuhan city in China.

The number of sample hospitals in the analysis model conforms to the basic principles of DEA method [10,11,12,59,71] as the following formula:

$$Z\;\geq \;\max\left\{3\left(x\;+\;y\right),\;\mathrm{xy}\right\}$$

where *x* denotes the input variables number, *y* denotes the output variables number and Z denotes the sample number used in DEA model. Sample hospitals can be divided into three categories based on differences in administrative subordination (Table 1). The geographical location of WH is illustrated in Figure 1.

### 2.5. Input–Output Variable Selection

In this study, the selection of input and output variables was guided by previous studies [7,8,9,10,11,12,13,14,30,31,32,33,34,35,36,37,38] and the Delphi method [72,73]. The reason for using Delphi was to keep social distance during COVID-19. The most commonly used indicators for evaluating hospital efficiency were screened out after literature search. Web of Science, PubMed, and China National Knowledge Infrastructure were searched. The search strategy consisted of combined terms of hospital, data envelopment analysis, efficiency, and productivity. 

**Figure 2:**
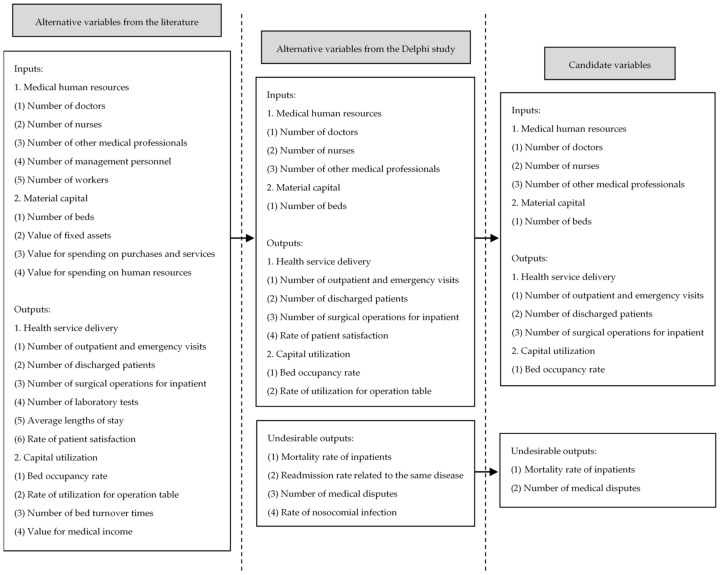
Flow chart for candidate variables selection of public hospital efficiency evaluation.

Next, Delphi technique was employed in this study. Five experts in hospital management and five professors in the field of Health Economics were invited to select candidate variables independently and anonymously from alternative indicators. The members of Delphi panel are from the following institutions: Dabieshan Medical Group (Huanggang, China), Tongji Medical College (Wuhan, China), Hainan Medical University (Haikou, China), and Hubei University (Wuhan, China). The whole process was conducted in November 2020 through the Tencent Meeting mobile application. All members responded to the designed questionnaire in three rounds. In the first round, all variables identified after review were submitted to the members of the Delphi team in the form of electronic files to determine the classification of variables (input variables, output variables, and undesirable variables). In the second round, a 5-point Likert scale questionnaire was used for scoring each variable based on its importance in the efficiency evaluation of tertiary hospital [72]. Each member of the Delphi panel rated variables on a scale of one (not important) to five (very important). Table 2 presents the Delphi findings of each variable in this round. In the third round, we provided the experts with the opportunity to think and rate again to make the results consistent. Results of the questionnaire were analyzed by median score and Interquartile Range (IQR) [73]. Median was calculated for the importance of how each item was scored. IQR was used to assess the success of the members’ agreement. Finally, indexes for four inputs, four outputs, and two undesirable outputs were selected. Figure 2 demonstrates the selection process of the variables. 

Regarding the input variables, most scholars consider both medical human resources and material capital as the main aspects in public hospitals [33,36,37,38,39,40]. Three variables were concentrated on human resources: Number of doctors (NoD), including full time equivalent (FTE) doctors and assistant doctors; Number of nurses (NoN), namely FTE registered nurses; Number of other medical professionals (NoOMP), consisting of FTE pharmacists, laboratory technicians, and other medical staff. Meanwhile, given the fact that extra and temporary beds are common in Chinese hospitals, Number of average actual open beds (NoAAOB) was used on behalf of material capital input. The statistical approach of NoAAOB was calculated through dividing the actual available bed days by the number of days in a year.

In terms of output variables, relevant research to hospital efficiency is prone to define diagnosis and treatment as the main output of hospitals [74,75]. Four variables have been considered in this research: Number of outpatient and emergency visits (NoOEV); Number of discharged patients (NoDP); Number of surgical operations for inpatient (NoSOI), and bed occupancy rate (BOR). BOR was calculated by dividing actual occupied bed days by actual available bed days in a year. At the same time, Mortality rate of inpatients (MRoI) and Number of medical disputes (NoMD) as undesired output were considered. MRoI was calculated by dividing the number of death cases of inpatients by the number of inpatients visits in a year.

### 2.6. Robustness of Estimation

To avoid the possibility of bias in index selection and to test the robustness of results in the estimation, three models depending on different variables were adopted. This is considering the verification of the sensitivity of technical efficiency changing in composition. Since the optimal production frontier is distinct from diverse variable selection, results of each DMU will be discrepant too [8,9]. Moreover, the efficiency frontier is only a measurement, exerting no influence on true reflection of each DMU’s relative technical efficiency. Therefore, the results of multiple models are not only contradictory but also mutually verifiable, reflecting various aspects of reality and thus providing strong evidence for decision-making. Meanwhile, the advantage of the procedure is its suitability of measuring limited samples. 

Model A was defined as the basic model incorporating the essential efficiency function. It was the most used in research of hospital efficiency, irrespective of undesirable outputs. Two additional models (Model B and Model C) performed as auxiliary tools. In these two models, normal variables diminished in comparison with Model A (Table 3) based on the importance of variables in findings of Delphi method. According to Seiford L’s study [76], undesirable outputs were dealt with in this research as follows: Model B treated undesirable outputs as normal inputs. [59] Model C used linear transformation to deal with undesirable outputs. [59,76] Both were designed to strengthen the robustness of the basic model’s results. 

### 2.7. Analysis Tools

Sample data were analyzed by SPSS (Version 19.0, IBM Corp, New York, NY, USA) for statistical description. MaxDEA Ultra (Version 7.9, Realworld Corp, Beijing, China), a powerful piece of DEA software that contains thousands of DEA models for various combinations was therefore employed to perform the DEA and Malmquist Index. Furthermore, a sensitive analysis was performed by bootstrapping with 2000 replications, providing the corrected efficiency indices of the analyzed model. Meanwhile, the significance of results was contrasted by Kruskal–Wallis test.

## 3. Results

### 3.1. Description of DMUs

**Figure 3:**
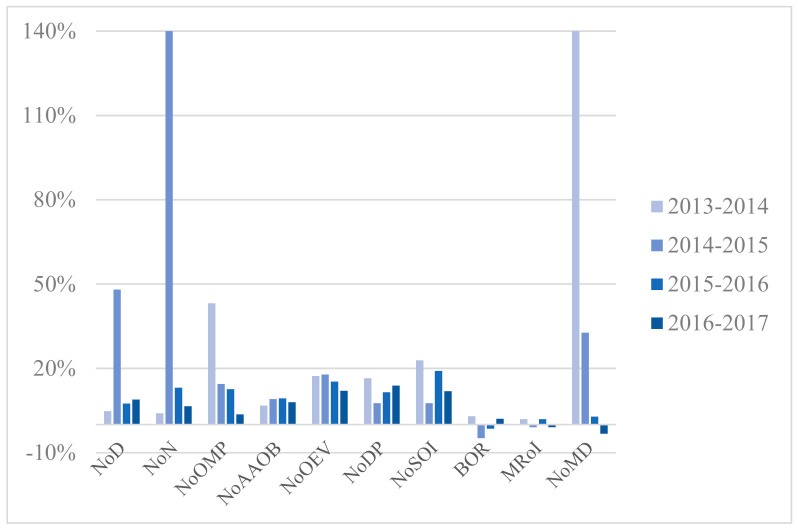
Annual growth rates of variables, PH.

**Figure 4:**
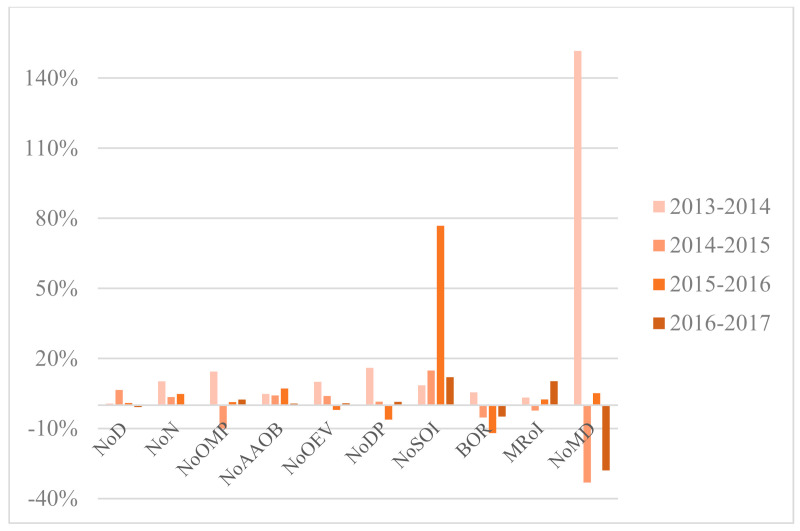
Annual growth rates of variables, OH.

**Figure 5:**
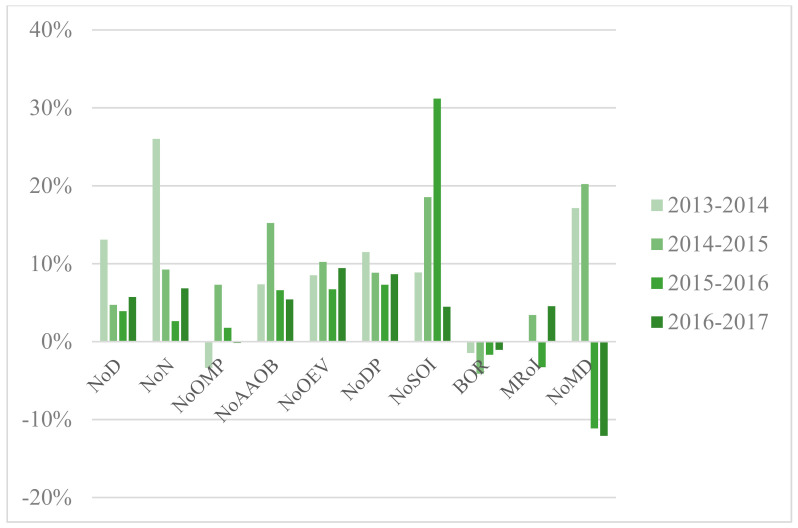
Annual growth rates of variables, MH.

Situated in central China, Wuhan is the largest city in this region with 10.89 million permanent residents and 81.16 million hospital visits in 2017 [77,78]. As one of the fastest-growing cities in China, Wuhan has 354 hospitals, 61 of which are tertiary hospitals [78]. The annual growth rates of variables regarding the three types of hospital are illustrated in Figure 3, Figure 4 and Figure 5. Appendix A presents yearly description statistics of input–output variables spanning from 2013 to 2017. 

Overall, the average of NoD in sample hospitals increased by 45.81% and NoN increased by 93.30% from 2013 to 2017. As for NoOEV, the growth rate reached 55.88%. Both the mean and SD of the inputs and outputs indicators grew year by year except for some fluctuations in NoOMP and BOR. However, the value of SD manifested huge disparities among sample hospitals, especially in variables such as NoOEV and NoDP. That is to say, the growth and diversity of indicators showed market potential but unbalanced development of tertiary public healthcare sector in WH at the same time.

On the one hand, the average of NoD, NoN, and NoOMP in PH achieved 1016.14, 1880.71, and 415.14 respectively in 2017, surpassing Municipal Hospitals (MH) and Other Hospitals (OH). That signified PH possessed more resources in medical personnel compared with MH and OH. Furthermore, the average of NoAAOB in Provincial Hospitals (PH), MH, and OH respectively stood at 2805.00, 1385.42, and 1000.33 in 2017. From the statistics, it is clear that PH scored significantly higher on average of NoAAOB than MH and OH due to ampler resources of inpatient beds. 

On the other hand, the mean of NoOEV and NoDP in PH, MH, and OH experienced a remarkable increase over the period. Meanwhile, the average NoSOI differed greatly among PH, MH, and OH and grew incrementally over the time horizon. However, the variable BOR showed a slight fluctuation and presented a relatively stable tendency between 2013 and 2015. 

As for undesirable outputs, the average of MRoI for each type of hospital fluctuated within their respective ranges. The mean of NoMD among three kinds of sectors had an upward trend from 2013 to 2016, which reminds us that communication and contact between doctors and patients in hospital evolvement should be paid attention to.

### 3.2. Technical Efficiency Comparison

Generally, the average efficiency in PH and MH showed an increasing tendency over the period. However, regarding OH, the reverse seems to be the case. Table 4 summarized the mean and Standard Deviation (SD) of original scores for CCR model orientated to outputs among Model A, Model B, and Model C. The analysis results of three models showed that public hospitals governed by municipal administration achieved higher mean scores than those affiliated with other levels of administrations. Under different subordinate relations, original score of sample hospitals did not significantly differ from 2013 to 2015 based on three models. However, the scores in 2016 and 2017 were statistically significant. 

Moreover, as we can see in Table 5, the results with bootstrapping in 2000 replications indicated an overall decline of efficiency score under three levels of affiliations owing to the corrected efficiency indices of the analyzed model. As for the mean scores with bootstrapping in Model A, Model B, and Model C, the efficiency value of MH was also higher than that of PH, and OH was the lowest over the period, except result of Model C in 2015 (PH scored the highest). Under different subordinate relations, score with bootstrapping of sample hospitals also did not significantly differ from 2013 to 2015 based on three models. However, the scores in 2016 and 2017 were statistically significant, except result of Model B in 2016. 

**Figure 6:**
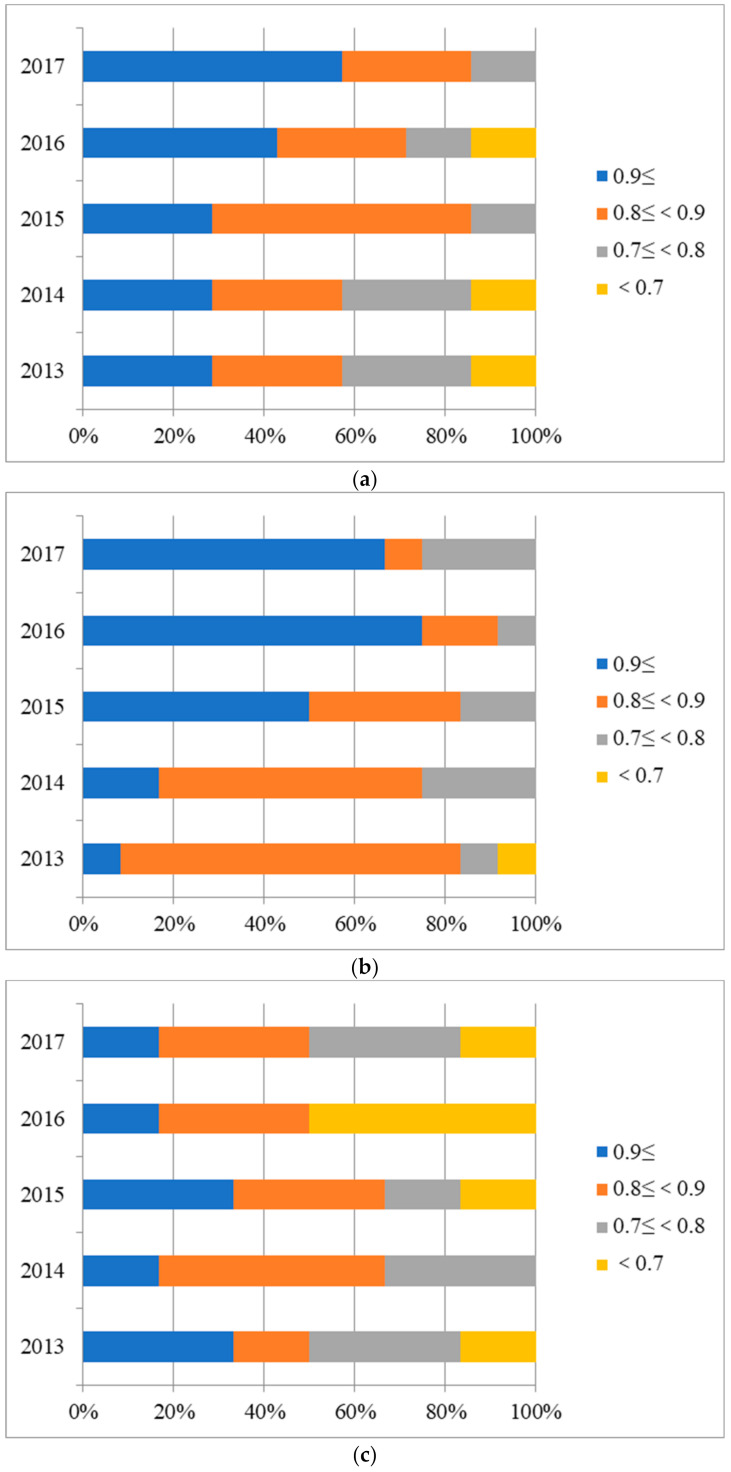
Distribution of the scores with bootstrapping of public hospitals based on Model A: (**a**) Provincial hospitals; (**b**) Municipal hospitals; (**c**) Other hospitals.

Next, distribution analysis of scores for hospitals under three affiliations were conducted. Given the limited space of the paper, only results of Model A with bootstrapping are shown in Figure 6. As we can see from it, some hospitals were apparently operating inefficiently and there were obvious differences in distribution of values among hospitals affiliated under different subordination.

As described in Figure 6, the distribution of scores with bootstrapping among PH, MH, and OH was exhibited in four ranges. In the first place, the distribution of score (>0.9) for PH kept increasing over the period, accounting for 57.14% in 2017 from Figure 6a. In addition, there was no efficiency value (<0.7) in the distribution of 2015 and 2017, reflecting a steady improvement of efficiency value in PH. Meanwhile, Figure 6b reported a remarkable efficiency growth in MH whose value (>0.9) made up a significant share of 75.00% in 2016 and the value (<0.7) has disappeared since 2013. Moreover, it can be found in Figure 6c that in OH, the proportion of value (>0.9) and value (0.9>, ≥0.8) was respectively 16.67% and 33.33% in 2017, and a downward trend took place. 

### 3.3. Mamlquist Index Change and Decomposition

The results for productivity change levels via MI with CRS from 2013 to 2017 are reported in Table 6. As shown in the table, the geometric means of DMUs indicated a slight increase of 0.54% in productivity from 2015–2016 but a decrease in 2013–2014, 2014–2015, and 2016–2017 (1.52%, 12.01%, and 0.07%, respectively). The OH was the only category that has been diminishing constantly in productivity from 2013 to 2017, while PH and MH showed fluctuations over the period. 

Focusing on the rise and fall of productivity variation between the Technological Change (TC) and Technical Efficiency Change (EC) in DMUs, we observed that the increase of MI was caused by the ascent of either EC or TC at different periods. Thus, it is challenging to figure out whether there is a clear demonstration of changes in public hospital productivity that can be attributed to EC or TC. Additionally, under different subordinate relations, MI, EC, and TC in sample hospitals did not significantly differ based on Model A, except the results for MI and TC in 2013–2014, MI and EC in 2016–2017. 

## 4. Discussion

Public hospitals serve as the center of the healthcare delivery chain, playing an essential role in the health service system, especially in a socialist country such as China. Currently, Chinese public hospitals occupy 95% of national healthcare resources and undertake the major responsibility of state health security [16,23]. However, the inadequacy and lack of access to affordable healthcare have been lingering in the public medical sector [19,21]. Although this phenomenon is caused by many factors, the root cause lies in the uneven distribution and usage of medical resources. Supposing the efficiency of public medical institutions is fairly low, it is still hard for the government to solve this dilemma even if fiscal subsidies are increased. Therefore, evaluating the efficiency of hospitals cannot be ignored while carrying out the PPHR. Reforms in the government and management system of public hospitals are still evolving globally. It is indisputable that the hospital sector is characterized by huge differences in scale, type, function, affiliation, and integrated performance in China. Fundamentally, how to use limited resources to improve efficiency and maximize the economic and social benefits of hospitals is the top priority for policymakers. The empirical results of our research are as shown below.

First, efficiency variance of public hospitals under different affiliations has already been shown gradually. The tendency of efficiency growth curve indicates that the differences in public hospitals affiliated with different levels of administrative organs are likely to grow over time. To be specific, it is clear that MH and PH have seen an obvious efficiency improvement between 2013 and 2017. The great majority of MH and PH achieved efficiency gains, accounting for most of the highest efficiency values of DMU cluster. However, OH had lost efficiency over the research panel and took on a decreasing trend, posing a tremendous challenge to the improvement of overall efficiency in public health facilities. Part of the reason for this result is efficacy of healthcare delivery in MH and PH benefits from the PPHR to some degree since 2014, because the policy circumstance of medical care in WH was relatively stable over the period except during PPHR’s implementation. Generally, this reform policy package may exert active effects on efficiency of MH and PH. However, it is still an issue to be explored as for why PPHR did not bring improved efficiency of OH. To ensure government stewardship in effectively leading public hospital system through next phase of reform, relevant measures should start with OH and pilot initiatives need to be clearly defined and explicitly funded to assist OH in achieving a better performance.

Second, MH achieved better results of average efficiency scores than those of PH and OH, whether before or after the implementation of PPHR. To the best of our knowledge, the higher efficiency of hospitals affiliated with municipality, as compared with those governed by province and under other affiliations, may be attributed to better governance and organization structure, such as the establishment of the Urban Medical Association. This kind of flexibility is in line with the form of localized management of medical resources. In addition, the input of MH resource, such as site size, medical facilities, and human resources, is considered more from the aspect of city instead of region. However, PH input is usually considered from the aspect of province and OH considered from community. These reasons may have caused the low efficiency of PH and OH in city. Hence, to enhance the use level of input resources and reduce wastes, further reform measures should be taken to restructure the input–output patterns of public health facilities according to the functional localization. To improve the efficiency of tertiary general public hospital clusters in WH, the quantity and location of PH and OH need to be judged and reconsidered.

Third, the distribution analysis of efficiency scores showed that most of the inefficient DMUs came from OH. There is no doubt that OH has the highest degree of autonomy and independence among the three types of hospitals, but it also produced the most ineffective values. The reason for this is probably that the management mode in the OH cluster was more diversified than PH and MH. Therefore, relevant heterogeneity existed in OH and it should be taken into consideration. In China, OH (i.e., hospitals governed by state-owned enterprises, universities, social groups, and social organizations) vary in organizational structure, financial affairs, and management mechanism. This kind of decentralization gives enough responsibility and autonomy to the healthcare facilities, such as management trusteeship and service outsourcing. However, decentralization may have negative repercussions [79]. Due to the intervention of private capital, it is reasonable to suppose that when hospitals are autonomous and independent, they may pursue their specific interests in the first place by placing barriers to the implementation of measures related to regional or national priorities (e.g., system reform or project implementation) instead of considering how to improve efficiency [79,80,81]. Although autonomy in healthcare has been successful in some European countries, we still doubt whether autonomy and independence can improve the efficiency of Chinese public hospitals. However, it is still a very interesting issue whether to adopt a centralized or decentralized governmental practice when supervising public healthcare sectors. We look forward to follow-up evidence from China. Therefore, more efforts should be made in OH to enhance its efficiency and reduce disparities among public hospitals affiliated with different levels of administration varying in efficiency. It is advisable of the government and organizations to examine the least efficient OH to remedy the prevailing inefficiency. Measures may include reconsidering the number of facilities and their distribution, enhancing efficiency, and reducing duplication by closing or scaling down hospitals with performance values below a certain threshold. Meanwhile, multiple policy mechanisms could be used consistently to put pressure on hospitals to contain costs and use resources more effectively.

Fourth, the results of MI and its decomposition cannot attest to differences among hospitals under distinct affiliations during the research panel. However, as we can notice from the MI curve for each sector, most productivity indices were in a regression state (<1). This reminds us that we must continue to pay attention to the productivity change of public healthcare facilities. Although public institutions will remain a major supplier in Chinese healthcare system, private medical market in which doctors can practice and patients can obtain reimbursement with healthcare insurance could grow rapidly over the next ten years due to limitations eased by government. The private healthcare facilities in China should be encouraged to play a significant part by transferring pressure to the public health sector, thus promoting efficient performance and fostering a benign competition environment.

Finally, the results by bootstrapping in 2000 replications are indicative of a general decline of the efficiency score compared with original ones, reflecting an optimal precision in the assessment. According to the research conducted by Angeliki, the bootstrapping method allows for the conclusion whether a result indicates true states or is a coincidence due to sampling variation [47]. Specifically, as an effective way to avoid possibility of bias in the estimation, the bootstrapping method can help break the bottleneck by repeated sampling to amplify the number of DMUs, to make the estimated efficiency scores much closer to the real ones. Also, bootstrapping technique could correct the biased SD caused by dependency in the panel data used in the study. For those reasons, bootstrapping method is strongly recommended when applying DEA approach in hospital efficiency estimation. 

## 5. Conclusions

This paper combined multiple approaches such as the Bootstrap-DEA and Malmquist method, and analyzed the disparity of efficiency scores and changes of productivity among public hospitals affiliated with different levels of administration in WH, through using panel data collected from 2013 to 2017. Our findings provided preliminary evidence that differences in public hospitals’ operation efficiency resulting from different administrative affiliations have emerged and are increasing year by year with the progress of PPHR. Based on DEA model, the average efficiency of MH ranked first among the three affiliations, and OH constituted the majority of inefficient DMUs. The higher efficiency of MH may be attributed to better governance and organization structure. Meanwhile, no evidence showed that there is a difference in productivity among hospitals under different affiliations. Moreover, we surmised that the PPHR, to a certain degree, may have exerted a positive influence on promoting the efficiency of PH and MH, but not OH. Thus, more effective measures should be initiated to help OH to alter their inefficient status. The challenge of boosting public hospital efficiency requires the implementation of reform in a more consistent, coordinated approach, to reengineer the process, especially in the administrative affiliations. 

## 6. Strengths and Limitations

There have been previous studies evaluating public hospitals’ efficiency from the aspects of service capacity and facility type, but we explored a unique perspective—administrative affiliations. However, there are still some limitations in the study. First, based on the defects of DEA approach, the lack of revision for case-mix and evaluation for absolute efficiency implies that the outcome of our research must be interpreted scrupulously and served only as a window into the performance of Chinese public hospitals. Second, although this study employed a significance test and treated all tertiary general public hospitals in WH as a clustered sample of PPHR, there may be variance of certain concern due to limited cluster. Further studies are required to incorporate massive intercity samples to gain a comprehensive view of performance in the cluster cities of PPHR. Third, with the rapid development of China’s economy, the impact of environmental factors on the efficiency of medical institutions (e.g., GDP per capita) could be considered in future studies by using methods such as Four-Stage DEA. 

## Figures and Tables

**Table 1:** Category of public hospitals based on differences in administrative subordination in WH.

Category	Affiliated with and Governed by	Ownership	Observations
Provincial Hospitals	Provincial government	Government agencies	7
Municipal Hospitals	Municipal government		12
Other Hospitals	State-owned enterprises, Universities, Social groups, or Social organizations	Public institutions	6

Notes: Category of public hospitals is based on Wuhan Health and Family Planning Yearbook (2014–2018).

**Table 2:** Delphi results of candidate variables (2nd round).

Variables	Frequency	Median	Interquartile Range
Inputs			
Number of doctors	10	5	0
Number of nurses	10	5	0
Number of other medical professionals	10	4.5	1
Number of management personnel	9	4	1
Number of workers	8	2	0.25
Number of beds	10	5	0
Value of fixed assets	9	3	1
Value for spending on purchases and services	9	3	1
Value for spending on human resources	9	4	3
Outputs			
Number of outpatient and emergency visits	10	5	0
Number of discharged patients	10	5	0
Number of surgical operations for inpatient	10	4.5	1
Number of laboratory tests	10	3	0.75
Average lengths of stay	8	3.5	2.25
Rate of patient satisfaction	10	4	2
Bed occupancy rate	10	5	0
Rate of use for operation table	10	4.5	2
Number of bed turnover times	9	4	3
Value for medical income	8	3	3.25
Undesirable outputs			
Mortality rate of inpatients	10	5	0.75
Readmission rate related to the same disease	9	5	0
Number of medical disputes	9	5	3
Rate of nosocomial infection	10	3.5	2.75

**Table 3:** Model specifications.

Indicators	Variables *	Definition	Unit	Model A	Model B	Model C
Inputs	NoD	Number of FTE doctors and assistant doctors	Number	X	X	X
	NoN	Number of FTE registered nurses		X	X	X
	NoOMP	Number of other FTE medical professionals		X		
	NoAAOB	Number of average actual open beds		X	X	X
Outputs	NoOEV	Number of outpatient and emergency visits	Number	X	X	X
	NoDP	Number of discharged patients		X	X	X
	NoSOI	Number of surgical operations for inpatient		X	X	
	BOR	Bed occupancy rate	Percentage	X	X	X
Undesirable Outputs	MRoI	Mortality rate of inpatients	Percentage		X	X
	NoMD	Number of medical disputes	Number			X

Notes: 1. * Variable statistic was conducted by staff from local Health Administration at the end of the year. 2. Abbreviation: FTE, Full Time Equivalent. 3. X indicates that the variable is included in the model.

**Table 4:** Scores for DEA _CRS_ (CCR model) orientated to outputs.

Panel	Original Scores	2013	2014	2015	2016	2017
Model A						
PH	Mean	0.8899	0.8922	0.9244	0.8814	0.9304
	SD	0.1284	0.1185	0.0797	0.1267	0.0747
MH	Mean	0.9323	0.9242	0.9437	0.9809	0.9514
	SD	0.0925	0.0885	0.0793	0.0463	0.0794
OH	Mean	0.8726	0.8658	0.8586	0.8131	0.8304
	SD	0.1014	0.075	0.1268	0.1450	0.0939
Kruskal–Wallis test	*p* value	0.375	0.332	0.311	0.033 **	0.050 *
Model B						
PH	Mean	0.8686	0.8623	0.9131	0.8689	0.9073
	SD	0.1243	0.1260	0.0893	0.1314	0.0825
MH	Mean	0.9203	0.9181	0.9362	0.9544	0.9420
	SD	0.1055	0.0957	0.0937	0.0741	0.0826
OH	Mean	0.8687	0.8630	0.8575	0.7878	0.7536
	SD	0.0995	0.0782	0.1257	0.1412	0.0535
Kruskal–Wallis test	*p* value	0.376	0.287	0.248	0.043 **	0.003 ***
Model C						
PH	Mean	0.8266	0.8565	0.8989	0.8439	0.8877
	SD	0.1256	0.1336	0.0950	0.1397	0.0947
MH	Mean	0.8902	0.9035	0.9106	0.8809	0.9063
	SD	0.1372	0.1069	0.1166	0.1306	0.1007
OH	Mean	0.7771	0.8272	0.8025	0.7064	0.7347
	SD	0.1189	0.0524	0.1054	0.1259	0.0741
Kruskal–Wallis test	*p* value	0.303	0.360	0.205	0.075 *	0.012 **

Notes: 1. Data source: Own elaboration. 2. Single asterisk (*) denotes significant differences at 90%, double asterisk (**) denotes significant differences at 95% and triple asterisk (***) denotes significant differences at 99%. 3. Abbreviation: CRS, Constant Returns to Scale; CCR, Charnes Cooper Rhodes; SD, Standard Deviation; PH, Provincial Hospitals; MH, Municipal Hospitals; OH, Other Hospitals.

**Table 5:** Scores with Bootstrapping for DEA _CRS_ (CCR model) orientated to outputs.

Panel	Scores with Bootstrapping	2013	2014	2015	2016	2017
Model A						
PH	Mean	0.8210	0.8243	0.8642	0.8262	0.8790
	SD	0.1051	0.0982	0.0615	0.1068	0.0592
MH	Mean	0.8516	0.8534	0.8761	0.9108	0.8797
	SD	0.0643	0.0655	0.0631	0.0389	0.0632
OH	Mean	0.8223	0.8261	0.8175	0.7678	0.7812
	SD	0.0921	0.0710	0.1180	0.1291	0.0741
Kruskal-Wallis test	*p* value	0.973	0.660	0.580	0.031 **	0.031 **
Model B						
PH	Mean	0.8011	0.7938	0.8555	0.8066	0.8441
	SD	0.1021	0.1039	0.0737	0.1087	0.0606
MH	Mean	0.8322	0.8437	0.8674	0.8767	0.8600
	SD	0.0750	0.0698	0.0760	0.0561	0.0644
OH	Mean	0.8116	0.8197	0.8163	0.7482	0.7068
	SD	0.0887	0.0729	0.1183	0.1365	0.0492
Kruskal-Wallis test	*p* value	0.963	0.482	0.677	0.137	0.005 ***
Model C						
PH	Mean	0.7387	0.7826	0.8386	0.7636	0.8196
	SD	0.0827	0.1066	0.0802	0.1109	0.0714
MH	Mean	0.7859	0.8261	0.8354	0.7901	0.8217
	SD	0.1026	0.0787	0.0964	0.1065	0.0748
OH	Mean	0.7160	0.7769	0.7579	0.6551	0.6850
	SD	0.0995	0.0497	0.0984	0.1251	0.0737
Kruskal-Wallis test	*p* value	0.359	0.320	0.225	0.086 *	0.014 **

Notes: 1. Data source: Own elaboration. 2. Single asterisk (*) denotes significant differences at 90%, double asterisk (**) denotes significant differences at 95% and triple asterisk (***) denotes significant differences at 99%. 3. Abbreviation: CRS, Constant Returns to Scale; CCR, Charnes Cooper Rhodes; SD, Standard Deviation; PH, Provincial Hospitals; MH, Municipal Hospitals; OH, Other Hospitals.

**Table 6:** Malmquist index of Model A with bootstrapping and its decomposition (2013–2017).

DMU	2013–2014			2014–2015			2015–2016			2016–2017		
	MI	EC	TC	MI	EC	TC	MI	EC	TC	MI	EC	TC
PH1	1.2098	0.9965	1.2141	0.5149	1.017	0.5063	1.0106	1.0056	1.005	0.9666	1.0227	0.9451
PH2	1.175	0.999	1.1761	0.4263	1.0271	0.4151	1.0606	1.0079	1.0523	1.0517	1.0232	1.0279
PH3	1.0296	1.0078	1.0216	0.55	1.0077	0.5458	1.0771	1.0048	1.0719	1.099	0.9864	1.1142
PH4	1.2443	1.1465	1.0854	0.8828	1.1587	0.7619	1.1678	1.006	1.1609	1.1157	1.0987	1.0155
PH5	1.0468	1.0039	1.0427	0.7974	0.787	1.0131	0.853	0.8354	1.021	1.0355	1.267	0.8173
PH6	0.9203	0.9356	0.9837	1.0641	1.3503	0.7881	0.9144	0.9342	0.9788	1.0228	0.9948	1.0281
PH7	0.9689	0.9609	1.0083	1.0799	1.1106	0.9723	0.8391	0.8717	0.9627	1.1199	1.1335	0.988
PH GM	1.0787	1.0054	1.073	0.7173	1.0534	0.6809	0.9823	0.9497	1.0343	1.0574	1.0714	0.987
MH1	0.9823	1.0094	0.9731	0.8094	1.0295	0.7863	1.0541	1.0091	1.0445	1.031	0.9836	1.0481
MH2	0.9763	1.0209	0.9563	0.9596	1.0107	0.9494	1.0239	0.9862	1.0382	1.0306	1.0139	1.0165
MH3	1.0756	1.1153	0.9644	0.8792	0.9452	0.9302	1.6326	1.311	1.2453	1.0044	0.961	1.0452
MH4	1.0217	0.9569	1.0678	0.9084	1.115	0.8147	1.2535	1.0494	1.1944	0.8812	0.8364	1.0536
MH5	0.9778	0.9244	1.0578	1.0348	1.1076	0.9343	1.007	1.1747	0.8573	0.8681	0.8552	1.0151
MH6	0.9967	0.9964	1.0003	0.9581	1.0224	0.9371	0.7319	1.0238	0.7149	1.0352	1.0178	1.0171
MH7	0.9655	1.0628	0.9084	0.9865	1.0251	0.9624	1.045	1.0425	1.0024	1.0601	0.9878	1.0732
MH8	1.0169	0.9971	1.0198	0.9913	1.0109	0.9806	1.0038	0.9932	1.0106	0.9378	1.0027	0.9352
MH9	0.91	1.0433	0.8722	0.9814	1.0164	0.9656	0.9821	1.001	0.9811	0.9415	0.9842	0.9566
MH10	0.6845	0.9037	0.7575	1.0452	1.0276	1.0171	0.9718	0.9652	1.0068	0.9265	0.9414	0.9842
MH11	0.8545	1.0102	0.8459	0.9523	1.0193	0.9342	1.0316	1.0037	1.0278	0.9786	0.9974	0.9811
MH12	0.8191	1.0035	0.8163	1.0507	1.0046	1.0459	1.063	0.9851	1.0791	0.9491	1.0102	0.9395
MH GM	0.9338	1.0021	0.9318	0.9606	1.027	0.9354	1.0496	1.0415	1.0078	0.9685	0.9641	1.0045
OH1	1.0036	0.9819	1.0221	0.9633	1.0506	0.9169	0.8992	1.0324	0.8709	0.9405	0.8831	1.065
OH2	0.9674	0.9364	1.0331	1.2436	1.2583	0.9883	0.5745	0.6282	0.9144	1.1423	1.2667	0.9018
OH3	0.9186	1.0739	0.8554	0.7989	0.7966	1.0029	1.1747	1.1755	0.9993	1.197	1.1923	1.004
OH4	1.0925	1.1331	0.9642	0.9561	0.9884	0.9674	1.0093	1.0288	0.9811	0.8481	0.8509	0.9967
OH5	0.9504	0.9255	1.027	0.8688	0.9153	0.9491	0.8247	0.8512	0.9688	0.9358	1.0269	0.9113
OH6	0.9858	1.009	0.977	0.8523	0.9385	0.9082	1.436	1.012	1.4189	0.957	1.0111	0.9465
OH GM	0.9849	1.0073	0.9778	0.9372	0.9815	0.9548	0.9479	0.9367	1.0119	0.9961	1.0277	0.9692
GM	0.9848	1.0043	0.9806	0.8799	1.0231	0.86	1.0054	0.9894	1.0161	0.9993	1.0084	0.991
*p* value	0.083 *	0.967	0.022 **	0.250	0.394	0.133	0.744	0.285	0.375	0.074 *	0.042 **	0.512

Notes: 1. Data source: Own elaboration. 2. Single asterisk (*) denotes significant differences at 90% and double asterisk (**) denotes significant differences at 95%. 3. Abbreviation: DMU, Decision-Making Unit; MI, Malmquist Index; GM, Geometric Mean; EC, Technical Efficiency Change; TC, Technological Change; PH, Provincial Hospitals; MH, Municipal Hospitals; OH, Other Hospitals.

## Data Availability

The statistical data of the study used and analyzed were extracted from publications, a series of Wuhan Health and Family Planning Yearbook (2014–2018). Online purchase links for publications are available from the first author on request.

## Appendix A. Description Statistics of Input–Output Variables (2013–2017)

**Table A1:** Description statistics of input–output variables (2013–2015).

Category	Variables	2013				2014				2015			
		Mean	SD	Max	Min	Mean	SD	Max	Min	Mean	SD	Max	Min
PH	NoD	559.86	320.66	1256.00	169.00	586.57	366.17	1405.00	171.00	868.29	560.36	1626.00	175.00
	NoN	620.57	223.32	1066.00	295.00	645.57	244.72	1165.00	299.00	1560.86	1157.02	3240.00	328.00
	NoOMP	217.14	94.56	363.00	94.00	310.86	213.84	667.00	95.00	355.71	249.91	815.00	101.00
	NoAAOB	2040.86	1211.66	3841.00	549.00	2178.43	1271.57	4165.00	598.00	2376.57	1497.98	4989.00	698.00
	NoOEV	1,583,595.43	1,310,464.21	3,513,216.00	73,244.00	1,857,237.43	1,591,916.68	4,285,597.00	93,461.00	2,187,820.71	1,853,284.48	4,829,868.00	110,391.00
	NoDP	75,648.71	53,985.43	158,426.00	6553.00	88,139.57	64,529.73	176,898.00	8760.00	94,864.57	71,565.41	195,593.00	9667.00
	NoSOI	26,225.71	21,879.51	54,725.00	892.00	32,217.43	26,407.06	71,308.00	1312.00	34,674.00	28,571.04	77,946.00	1524.00
	BOR	105.99	16.34	130.07	82.43	109.12	15.91	140.13	87.62	103.81	18.71	140.11	74.80
	MRoI	1.03	0.54	1.43	0.39	1.05	0.57	1.91	0.36	1.04	0.56	1.88	0.39
	NoMD	16.00	19.40	48.00	1.00	38.43	47.72	136.00	1.00	51.00	30.63	98.00	11.00
MH	NoD	378.33	188.52	724.00	142.00	427.83	225.24	836.00	151.00	448.00	244.07	900.00	164.00
	NoN	546.58	332.67	1109.00	158.00	688.75	398.12	1564.00	305.00	752.42	464.75	1805.00	323.00
	NoOMP	167.75	68.99	311.00	67.00	162.08	60.45	261.00	54.00	173.92	83.67	386.00	64.00
	NoAAOB	996.75	453.16	1759.00	467.00	1070.08	557.69	2388.00	498.00	1232.92	807.19	3350.00	498.00
	NoOEV	704,016.92	567,613.12	2,275,892.00	147,964.00	763,964.25	592,834.91	2,298,258.00	148,587.00	842,138.00	642,011.47	2,314,905.00	184,662.00
	NoDP	39,792.00	24,311.46	84,019.00	13,093.00	44,367.17	30,035.10	115,482.00	13,650.00	48,287.67	35,511.95	142,465.00	13,416.00
	NoSOI	10,859.25	6867.98	23,372.00	2380.00	11,823.42	8611.21	32,269.00	2108.00	14,015.50	10,394.95	40,048.00	2531.00
	BOR	106.22	16.60	136.45	74.43	104.67	13.38	126.10	79.03	100.34	10.31	113.20	83.07
	MRoI	0.88	0.54	1.76	0.20	0.88	0.56	1.87	0.10	0.91	0.60	1.94	0.10
	NoMD	29.17	26.76	82.00	1.00	34.17	33.71	115.00	1.00	41.08	35.45	105.00	2.00
OH	NoD	295.67	93.43	452.00	146.00	297.67	88.91	420.00	181.00	317.00	79.56	416.00	187.00
	NoN	448.50	101.99	575.00	276.00	494.17	117.12	676.00	346.00	511.33	89.83	639.00	413.00
	NoOMP	134.50	51.54	245.00	83.00	153.83	31.47	193.00	111.00	138.50	42.72	198.00	97.00
	NoAAOB	850.00	184.81	1193.00	630.00	890.67	203.82	1296.00	652.00	927.50	176.87	1296.00	794.00
	NoOEV	440,459.83	87,654.22	590,961.00	323,152.00	484,304.50	199,808.36	721,723.00	191,235.00	503,293.67	192,961.04	801,667.00	247,233.00
	NoDP	25,620.33	10,981.27	44,043.00	13,992.00	29,703.33	13,458.20	55,468.00	14,688.00	30,149.83	12,670.13	54,310.00	17,021.00
	NoSOI	4781.33	3142.49	10,354.00	839.00	5186.17	3814.95	12,418.00	753.00	5953.83	3814.79	12,315.00	581.00
	BOR	97.69	11.14	114.77	83.44	103.06	13.41	118.88	79.36	97.60	16.88	115.00	68.69
	MRoI	1.23	0.35	1.53	0.45	1.27	0.26	1.52	0.72	1.24	0.28	1.44	0.66
	NoMD	23.25	21.85	61.00	1.00	58.50	106.53	316.00	1.00	39.12	21.34	65.00	6.00

**Table A2:** Description statistics of input–output variables (2016–2017).

Category	Variables	2016				2017			
		Mean	SD	Max	Min	Mean	SD	Max	Min
PH	NoD	933.00	602.59	1775.00	183.00	1016.14	648.98	1907.00	193.00
	NoN	1765.43	1351.86	4003.00	360.00	1880.71	1426.49	4226.00	381.00
	NoOMP	400.57	296.34	987.00	118.00	415.14	324.78	1054.00	117.00
	NoAAOB	2598.43	1683.93	5128.00	800.00	2805.00	1885.18	5461.00	798.00
	NoOEV	2,522,946.14	2,234,223.89	5,800,846.00	126,331.00	2,826,435.14	2,493,122.59	6,317,152.00	136,685.00
	NoDP	105,800.29	83,418.86	231,248.00	10,583.00	120,493.43	96,914.89	272,002.00	12,174.00
	NoSOI	41,287.57	34,350.85	97,017.00	2198.00	46,187.29	37,629.60	104,156.00	2805.00
	BOR	102.26	18.02	128.77	68.44	104.38	17.14	124.68	74.21
	MRoI	1.06	0.57	1.95	0.41	1.05	0.59	1.93	0.38
	NoMD	52.43	37.57	126.00	17.00	50.71	20.77	88.00	26.00
MH	NoD	465.50	262.81	1004.00	164.00	492.17	278.25	1088.00	173.00
	NoN	772.25	483.47	1921.00	339.00	825.08	523.99	2160.00	343.00
	NoOMP	177.00	73.92	298.00	71.00	176.67	70.55	317.00	85.00
	NoAAOB	1314.17	829.79	3378.00	499.00	1385.42	831.06	3369.00	497.00
	NoOEV	898,697.33	704,974.89	2,370,588.00	174,047.00	983,537.00	730,772.53	2,510,640.00	170,712.00
	NoDP	51,813.08	38,378.75	156,783.00	14,282.00	56,297.17	41,130.54	168,712.00	13,911.00
	NoSOI	18,386.33	14,840.15	45,257.00	2594.00	19,208.42	15,381.71	52,054.00	2627.00
	BOR	98.64	13.93	119.05	75.79	97.59	13.25	121.56	79.89
	MRoI	0.88	0.56	1.86	0.11	0.92	0.53	1.79	0.10
	NoMD	36.50	27.66	87.00	2.00	32.08	21.24	64.00	3.00
OH	NoD	319.83	75.01	419.00	191.00	317.00	74.49	421.00	199.00
	NoN	535.67	105.83	705.00	407.00	535.17	123.16	746.00	405.00
	NoOMP	140.33	32.72	190.00	103.00	143.67	34.34	187.00	100.00
	NoAAOB	993.67	196.20	1392.00	812.00	1000.33	179.18	1277.00	808.00
	NoOEV	493,043.00	220,460.27	796,440.00	196,035.00	496,947.67	214,440.90	808,992.00	194,081.00
	NoDP	28,265.00	10,539.60	45,706.00	15,597.00	28,663.67	10,005.96	43,535.00	15,543.00
	NoSOI	10,523.33	10,219.57	32,451.00	627.00	11,781.33	9927.97	32,232.00	544.00
	BOR	85.90	14.51	106.68	67.07	81.68	10.56	94.46	66.67
	MRoI	1.27	0.23	1.48	0.83	1.40	0.24	1.65	1.13
	NoMD	41.13	37.93	124.00	2.00	29.63	16.33	62.00	7.00

Notes: 1. Data source: Wuhan Health and Family Planning Yearbook (2014–2018). 2. Abbreviation: SD, Standard Deviation; PH, Provincial Hospitals; MH, Municipal Hospitals; OH, Other Hospitals.
